# Supplementary material for: Culturomics and Circular Agronomy: Two Sides of the Same Coin for the Design of a Tailored Biofertilizer for the Semi-Halophyte Mesembryanthemum crystallinum
Source: Plants (Basel). 2023 Jul 4;12(13):2545. doi: 10.3390/plants12132545 (PMC10346144; doi:10.3390/plants12132545)

Supplementary Information Table S1. Genera and species isolated using the TSA standard medium and the MA medium, indicating the compartments they were isolated from.

TSA MEDIUM				
**Genus**	**Number of isolates**	**Species**	**Number of isolates**	**Plant compartment**
Bacillus	27	*subtilis*	8	rhizosphere soil, roots
		sp.	5	roots, shoots
		amyloliquefaciens	4	roots
		pumilus	3	roots, shoots
		cereus	2	roots
		inaquosorum	1	shoots
		licheniformis	1	roots
		mycoides	1	roots
		siamensis	1	rhizosphere soil
		velezensis	1	roots
Pseudomonas	5	*gessardii*	1	shoots
		sp.	4	rhizosphere soil
Priestia	3	*aryabhattai*	2	shoots
		megaterium	1	shoots
Pantoea	3	*agglomerans*	2	rhizosphere soil
		alhagi	1	rhizosphere soil
Rosellomorea	3	*vietnamensis*	1	roots
		aquamaris	1	roots
		sp.	1	roots
Cytobacillus	1	*firmus*	1	roots
Glutamicibacter	1	*arilaitensis*	1	rhizosphere soil
Leclercia	1	sp.	1	shoots
Lysinibacillus	1	*fusiformis*	1	shoots
Mesobacillus	1	*foraminis*	1	roots
Microbacterium	1	*kitamiense*	1	shoots
**TOTAL NUMBER OF ISOLATES IN TSA MEDIUM**				**47**

MA MEDIUM				
**Genus**	**Number of isolates**	**Species**	**Number of isolates**	**Plant compartment**
Priestia	11	*aryabhattai*	8	rhizosphere soil, roots, shoots
		megaterium	3	rhizosphere soil, roots
Bacillus	10	*subtilis*	5	roots
		licheniformis	1	roots
		velezensis	1	shoots
		amyloliquefacien	1	shoots
		sp.	1	rhizosphere soil
Curtobacterium	5	*flaccumfaciens*	1	roots
		sp.	4	roots, shoots
Microbacterium	3	*kitamiense*	1	roots
		sp.	2	roots
Pseudomonas	3	*chlororaphis*	1	shoots
		sp.	2	rhizosphere soil
Staphylococcus	2	*cohnii*	1	roots
		warneri	1	shoots
Rossellomorea	1	sp.	1	roots
Cytobacillus	1	*oceanisediminis*	1	rhizosphere soil
Mesobacillus	1	*subterraneus*	1	rhizosphere soil
**TOTAL NUMBER OF ISOLATES IN MA MEDIUM**				**37**

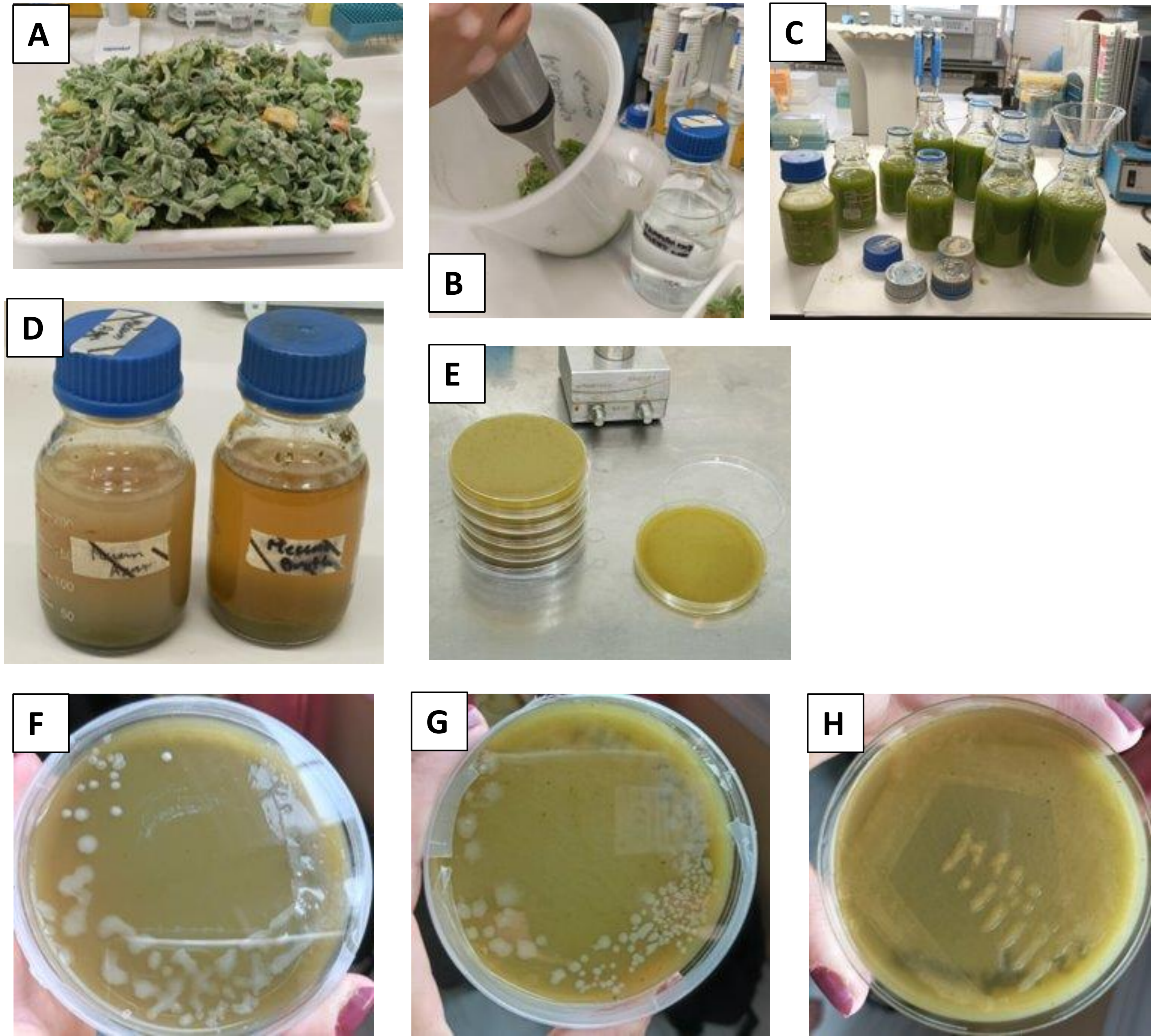

Supplementary Information Figure S1. A. Frozen tissues of *M. crystallinum* plants. B, C. Preparation of the medium MesemAgar (MA) with plant tissues and buffer using a mixer. D. Autoclaved media MesemAgar and MesemBroth. E. Plates of MesemBroth. F, G, H. Aspect of the strains MR4 (F), R5 (G) and MH8 (H) in medium MA.

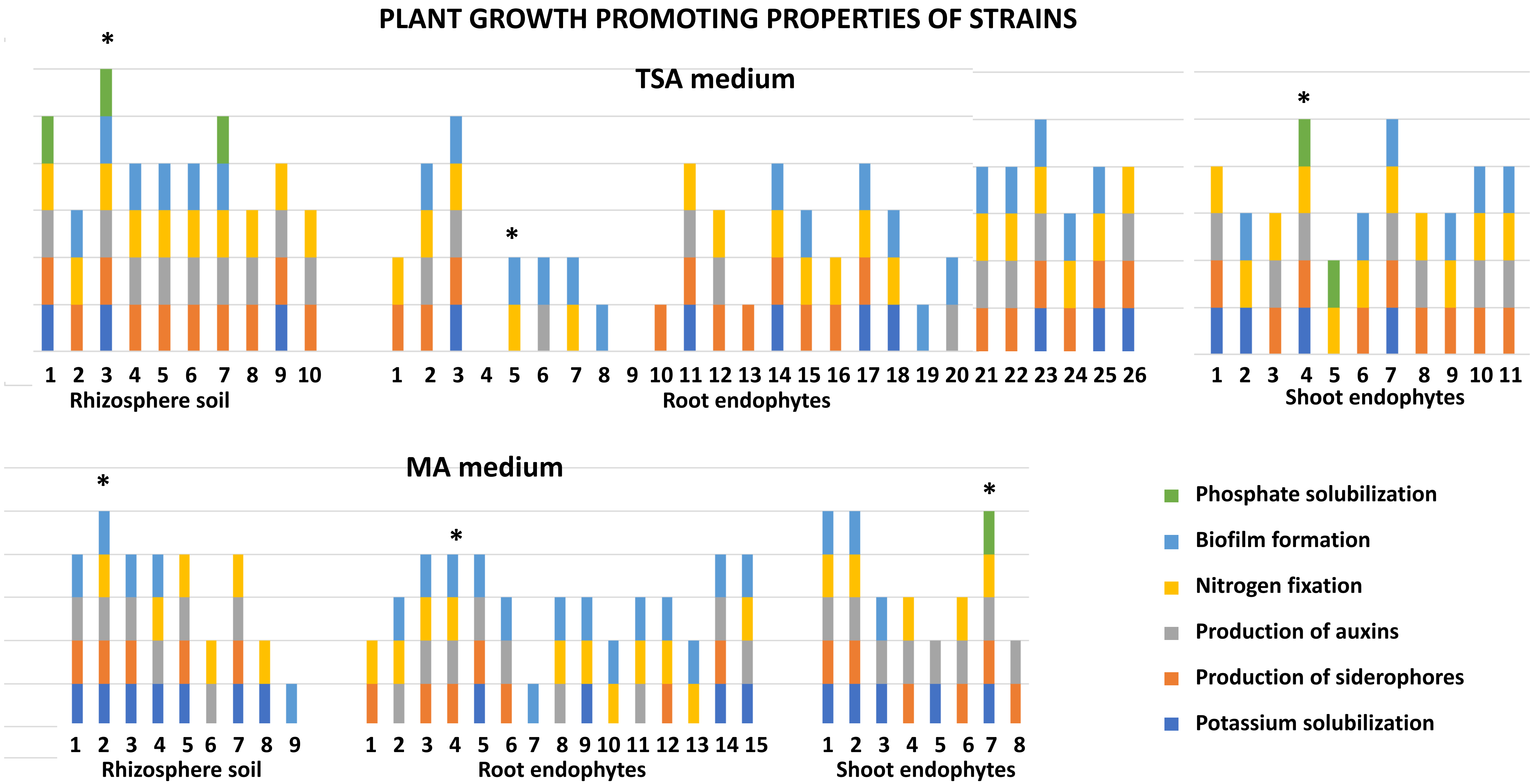

Supplementary Information Fig. S2: Plant Growth Promoting properties of the strains belonging to both collections. Each property has been assigned one color. The strains with the highest number of properties were selected (indicated with asterisks). Strain number 5 of root endophtes showing only two PGP activities was selected because the high number of extracellular activities (see Suppl. Inf. Fig. S3).

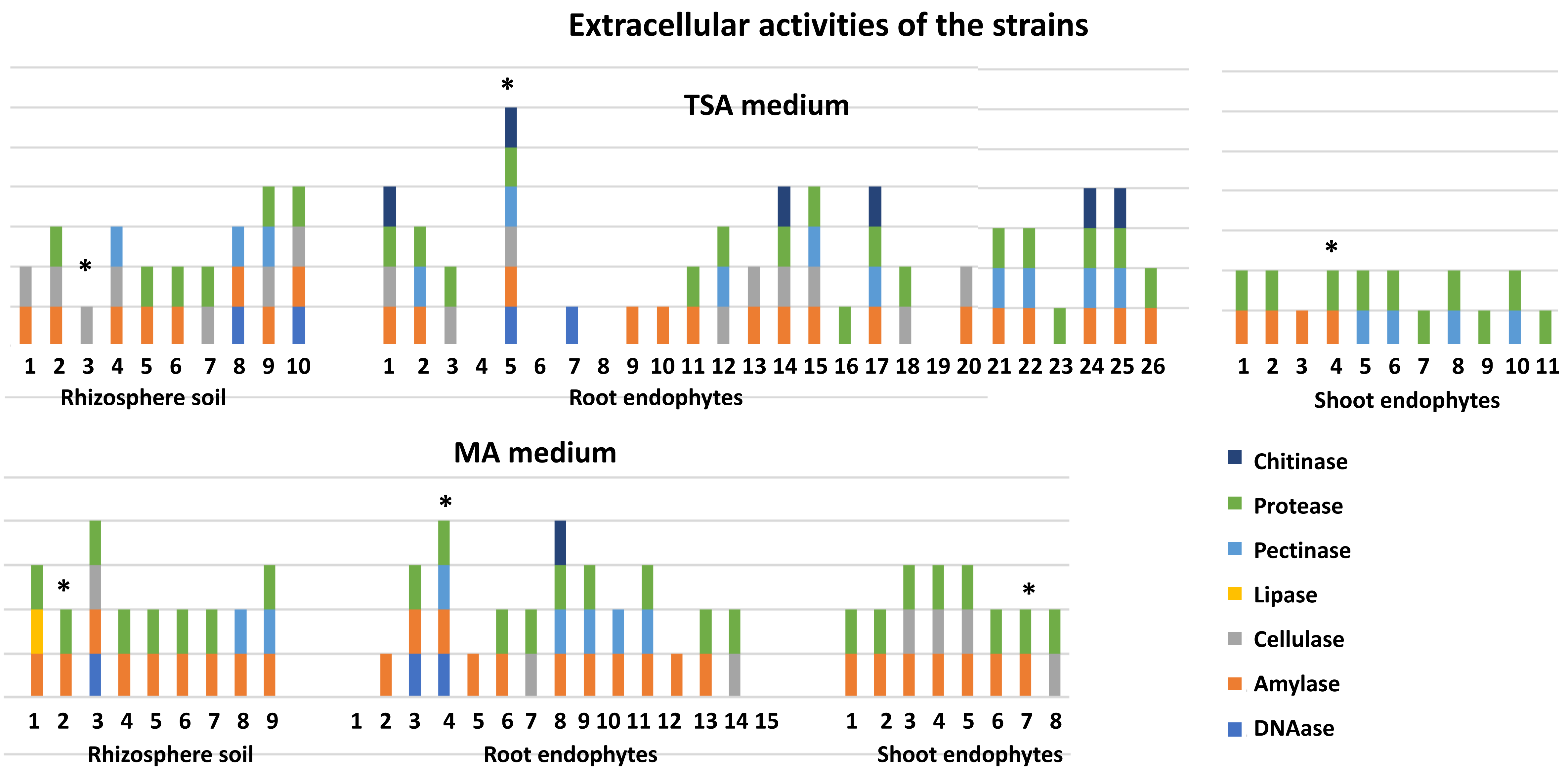

Supplementary Information Fig. S3: Extracellular activities of the strains belonging to both collections. Each property has been assigned one color. The strains indicated with asterisks were selected for the consortiums on the basis of PGP activities and extracellular activities. Strain number 3 of rhizosphere soil displaying only cellulase activity was selected because the high number of PGP properties (see Suppl. Inf. Fig. S2).

Percentage of traits by plant compartment and media

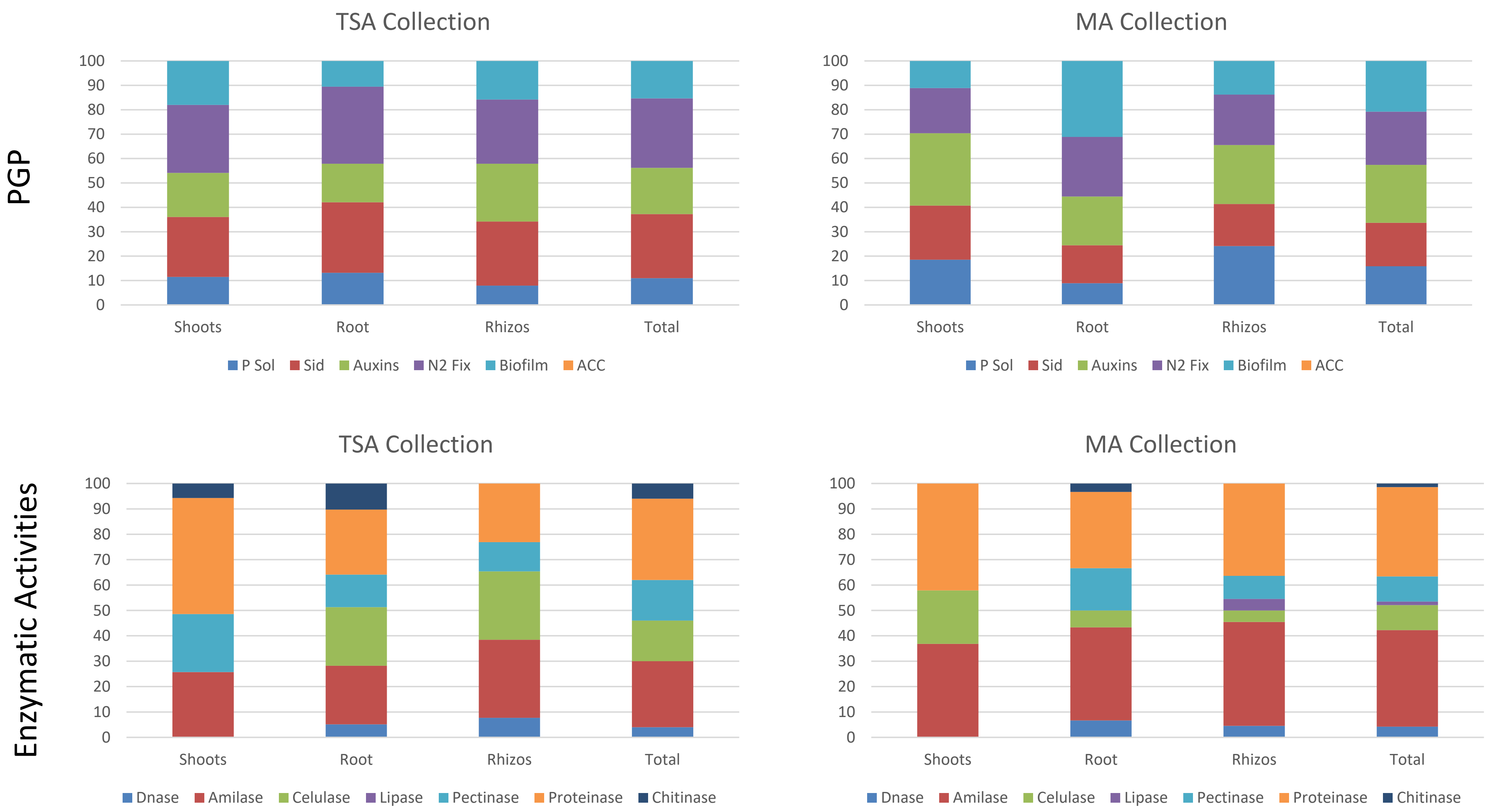

Supplement: Supplementary file 1 [file plants-12-02545-s001.zip › plants-2435872-supplementary.pdf]
